# Quality of Postoperative Recovery in Total Intravenous Anesthesia between Remimazolam and Propofol for Intraoperative Neurophysiological Monitoring: A Prospective Double-Blind Randomized Controlled Trial

**DOI:** 10.3390/jpm14040382

**Published:** 2024-04-02

**Authors:** Jiwon Lee, Dong Woo Han, Young Song, Jongyun Lee, Soyoung Jeon, Myoung Hwa Kim

**Affiliations:** 1 Department of Anesthesiology and Pain Medicine, Anesthesia and Pain Research Institute, Gangnam Severance Hospital, Yonsei University College of Medicine, Seoul 06273, Republic of Korea; belief705@yuhs.ac (J.L.); hanesth@yuhs.ac (D.W.H.); nearmyheart@yuhs.ac (Y.S.); jongyun27@yuhs.ac (J.L.); 2 Biostatistics Collaboration Unit, Department of Research Affairs, Yonsei University College of Medicine, Seoul 06229, Republic of Korea; jsy0331@yuhs.ac

**Keywords:** intraoperative neurophysiological monitoring, propofol, quality of recovery, remimazolam, total intravenous anesthesia

## Abstract

This study compared the overall postoperative recovery of patients who underwent total intravenous anesthesia with remimazolam or propofol, using the Quality of Recovery-15 questionnaire (QoR-15). Seventy-two patients who underwent spine surgery with intraoperative neurophysiological monitoring (IONM) were randomly categorized into the remimazolam group (group R) or propofol group (group P). On the first postoperative day, the QoR-15 scores for groups P and R were 114 and 112, respectively, indicating no significant difference (*p* = 0.691). Similarly, group–time interaction effects on QoR-15 scores were not significantly different. In the post-anesthesia care unit, the pain intensity at rest was notably higher in group P than in group R (3.0 [0.0] vs. 2.8 [0.5], respectively, *p* = 0.009). Although the intraoperative consumption of remifentanil was higher in group R (1452.4 µg vs. 2066.8 µg, respectively, *p* < 0.001), the intraoperative use of vasopressors was lower in group R (1705.6 µg vs. 286.1 µg, respectively, *p* < 0.001) compared to group P. Group R exhibited significantly lower variability in mean blood pressure over time compared to group P. Remimazolam was viewed as a promising intravenous agent for general anesthesia, showing potential to replace propofol in spine surgery with IONM, considering both recovery quality and intraoperative hemodynamic stability.

## 1. Introduction

Remimazolam, classified under the benzodiazepine drug category, acts as an ester-based agonist of the gamma-aminobutyric acid type-A (GABA_A_) receptor. Remimazolam has remarkable advantages over current benzodiazepines, such as rapid onset, short context-sensitive half-life, metabolism independent of hepatic or renal function due to tissue esterase, and production of an inactive metabolite [1,2,3,4]. These pharmacokinetic properties are similar to those of propofol, which is widely used to maintain general anesthesia as a continuous intravenous infusion [5]. Consequently, remimazolam has emerged as a safe option for continuous intravenous infusion and has been increasingly used as an anesthetic for sedation and general anesthesia. A few studies have compared postoperative recovery between remimazolam and propofol in general anesthesia, but these surgeries were relatively minor, and the results were inconsistent [6,7].

Even in the absence of complications, patients often experience discomfort during the recovery period after anesthesia or surgery, which can significantly affect their overall quality of life. Moreover, suboptimal postoperative recovery can lead to prolonged hospital stays and increased medical costs, potentially affecting long-term prognosis and patient satisfaction [8]. Consequently, anesthesiologists must carefully consider the use of adequate anesthetics and methods that enhance rapid and better quality of recovery, reduce perioperative discomfort and complications, and accelerate the resumption of daily activities. The Quality of Recovery-15 questionnaire (QoR-15), a simplified version of the QoR-40 questionnaire, allows patients to conduct self-assessments that help healthcare professionals gauge postoperative recovery effectiveness and address areas that may require improvement or attention [9,10,11].

Conventionally, propofol-based total intravenous anesthesia (TIVA) is considered as a superior anesthetic method in terms of quality of recovery (QoR) compared to other approaches. It is associated with low incidence of nausea and vomiting, which facilitates a smoother recovery from anesthesia [12,13]. These benefits position propofol-based TIVA as a favorable choice for promoting a positive postoperative experience and promoting overall patient satisfaction with the anesthesia process. Importantly, propofol is also used to maintain a stable and consistent signal of evoked potentials for intraoperative neurophysiological monitoring (IONM) [14,15]. However, concerns exist regarding injection pain and hemodynamic instability from propofol use, as well as the possibility of propofol infusion syndrome when administered intravenously over an extended period. Moreover, there is no antidote necessary for rapid recovery after anesthesia [16,17,18].

To date, no studies have compared the use of propofol and remimazolam using QoR questionnaires after TIVA for surgeries requiring IONM. Therefore, we conducted a study comparing postoperative overall recovery between remimazolam-based TIVA and conventional propofol-based TIVA, via the QoR-15, in patients who underwent spine surgery with IONM.

## 2. Materials and Methods

### 2.1. Participants Assignment

Our study, a prospective, double-blind, randomized controlled trial, was conducted at a tertiary hospital in adherence to the principles outlined in the 2013 Declaration of Helsinki. The study protocol received approval from the Institutional Review Board of Gangnam Severance Hospital (IRB No. 3-2021-0216), Yonsei University Health System, Seoul, Republic of Korea. Additionally, this trial was registered at clinicaltrials.gov (NCT04994704) before patient enrollment. All participating patients provided written informed consent.

The study population comprised individuals aged 20–70 years, with an American Society of Anesthesiologists physical status ranging from I to III, who underwent elective spine surgery necessitating IONM in the Department of Neurosurgery at our institution. Patients were recruited from December 2021 to July 2023. The exclusion criteria were as follows: patients with addiction or dependence on alcohol or psychotropic substances, those with benzodiazepine hypersensitivity or tolerance, and those with a body mass index exceeding 30 kg/m^2^.

The participants were randomly divided into two groups: the remimazolam group (group R) and the propofol group (group P), in a balanced 1:1 ratio. A computer-generated block randomization sequence was created by a researcher not involved in this study. Each assignment was concealed within sequentially numbered opaque envelopes. A researcher unveiled the envelope and allocated patients to their respective groups based on the enclosed assignment letter on the day of surgery. Throughout the investigation, patients, outcome researchers, and surgeons remained blinded to the patient group allocation.

### 2.2. Intervention

Patients arrived at the operating room without prior medication and underwent standard monitoring, including pulse oximetry, non-invasive blood pressure measurements, electrocardiography, and a SedLine^®^ sensor (Masimo Corp, Irvine, CA, USA) affixed to the forehead, which gauges the anesthetic depth, showing patient state index (PSI) value. For participants in group P, anesthesia induction was performed using 0.1 mg of glycopyrrolate, 40 mg of lidocaine, targeted controlled infusion (TCI) of propofol (Fresofol 2% injection 50 mL vial; Fresenius Kabi, Seoul, Republic of Korea) set at 3.0 ng mL^−1^ using the Schnider model [19,20], as well as TCI of remifentanil (Ultian injection 1 mg vial; Hanlim, Seoul, Republic of Korea) at 3.0 ng mL^−1^ via the Minto model [21,22]. Alternatively, in group R, anesthesia initiation involved 0.1 mg of glycopyrrolate, along with the commencement of a remimazolam (ByFavo; Hana Pharmaceutical, Seoul, Republic of Korea) infusion, spanning 6–12 mg kg^−1^ h^−1^ and maintained at 1.0–2.0 mg kg^−1^ h^−1^ after loss of consciousness [2,23], paired with a TCI of remifentanil at 3.0 ng mL^−1^ using the Minto model. A commercially available TCI pump (Agilia Connect; Fresenius Vial, France) was used in both groups.

Upon confirming the patient’s lack of response to verbal cues and absence of an eyelash reflex, 0.6 mg kg^−1^ rocuronium (Esmeron™, N.V. Organon, Oss, The Netherlands) was used as a neuromuscular blocking (NMB) agent for intubation, utilizing an 8 mm tube for men and a 7 mm tube for women. Additionally, the response of the adductor pollicis brevis muscle to train-of-four (TOF) stimulation of the ulnar nerve by a neuromuscular transmission module (M-NMT Module, Datex-Ohmeda Inc., Helsinki, Finland) was monitored every 15 min. Intraoperative monitoring included measurement of arterial pressure via the radial arterial line and body temperature via an esophageal stethoscope. The depth of anesthesia was adjusted to a mean arterial pressure (MAP) within 20–30% of the baseline value (minimum 65 mmHg) and a PSI of 25–50 points. To mitigate postoperative pain, an intraoperative injection of 20 mg nefopam and 1 g acetaminophen, alongside a 75 µg palonosetron intravenous administration for antiemesis, was implemented. Patient-controlled analgesia was prepared, including 10 µg kg^−1^ of fentanyl, 2 mg kg^−1^ of nefopam, and 0.6 mg of ramosetron, administered intravenously.

At the end of surgery, the anesthetic agent and remifentanil administration were discontinued, and NMB was assessed and reversed. The TOF was determined by reversal with administration of 0.07 mg kg^−1^ of neostigmine and 0.05 mg kg^−1^ of glycopyrrolate. Successful spontaneous breathing and recovery of consciousness led to extubation and patient transfer to the post-anesthetic care unit (PACU). Pain intensity during movement and rest was assessed using the 10-point numerical rating scale (NRS), and the use of rescue analgesics and their morphine equivalent consumption were documented. Complaints of nausea or vomiting and administered antiemetics were also recorded. Subsequently, at 6, 24, and 48 h postoperatively in the ward, pain intensity was monitored using the NRS, and use of rescue analgesics and antiemetics was evaluated.

### 2.3. Outcome Assessments

The primary outcome of our study was the total QoR-15 score measured on postoperative day (POD) 1. We used the Korean version of the QoR-15 questionnaire, which has been validated and found to be as effective and dependable as the original English version (Supplementary Digital Material: Supplementary Figure S1), to assess the comprehensive functional recovery of patients postoperatively. The questionnaire comprises the following five dimensions, encompassing inquiries regarding diverse aspects of postoperative overall recuperation: physical comfort (Q1, 2, 3, 4, 13), emotional state (Q9, 10, 14, 15), psychological support (Q6, 7), physical independence (Q5, 8), and pain (Q11, 12). Respondents rated each item on an 11-point scale (0–10), with higher scores indicating better attributes and lower scores indicating poorer attributes. The cumulative score ranged from a possible minimum score of 0 (indicative of the poorest recovery quality) to a potential maximum score of 150 points (signifying optimal recovery quality). During the study period, we conducted three QoR-15 survey sessions for patients at the following three time points: the day before surgery upon securing consent, on POD1, and on POD2. A researcher, blinded to the group assignments, administered questionnaires to ensure an unbiased approach.

We investigated hemodynamic parameters such as MAP, heart rate, peripheral oxygen saturation, and PSI values as secondary outcomes. These measurements were taken at six time points: baseline (before anesthesia), immediately after intubation, at the start of surgical stimuli, at 1 h after the start of surgery, at the completion of surgery, and at PACU discharge. We also assessed information related to anesthetic induction and emergence, such as duration of unconsciousness, instances of PSI values falling below 50, time taken for intubation after anesthetic administration, duration of consciousness return, instances of PSI values exceeding 50, and the time of extubation following anesthesia completion. Further evaluations of postoperative recovery profiles included NRS of pain intensity, postoperative nausea and vomiting, administration of rescue analgesics or antiemetics in the PACU and ward settings, duration of stay in the PACU, duration of postoperative hospitalization, and occurrence of postoperative complications.

### 2.4. Statistical Analyses

Our sample size was determined based on the results of a previous study [24]. The mean difference in the total QoR-15 score exceeded 13 points, signifying an improved postoperative recovery of health status as perceived by the patients. Notably, for intermediate-risk surgeries, which include procedures such as cervical spine surgery, the mean (±standard deviation) total QoR-15 score on POD1 was 114 (±18) points [24]. Consequently, we estimated that a sample size of 34 patients per group would be necessary to achieve a statistical power of 90% at a significance level (alpha) of 0.05. Accounting for a potential dropout rate of 5%, we ultimately enrolled a total of 72 patients, dividing them into two groups of 36 patients each.

Prior to performing the analysis, we verified the normal distribution of patient characteristics and perioperative variables using the Shapiro–Wilk test. Differences between groups were evaluated using an independent two-sample t-test for continuous variables and the Chi-square or Fisher’s exact test for categorical variables. Categorical variables, more than 20% of cells with an expected frequency of less than 5, were analyzed using the Fisher exact test, and those with less than 20% were analyzed with the Chi-square test. For an in-depth analysis, we employed linear mixed models and generalized estimating equations, incorporating an unstructured covariance matrix. These methodologies were designed to accommodate continuous and binary outcomes with repeated measurements. Our analysis included an assessment of the impact of the anesthetic agent on each postoperative outcome across groups at specific time points. This involved a comprehensive examination of the following aspects: (1) between-group distinctions (group effects), (2) within-group fluctuations from baseline (time effects), and (3) intergroup variances in changes from baseline (group–time interaction effects). Statistical analyses were performed using SAS software (version 9.4; SAS Institute, Cary, NC, USA). Statistical significance was established at a two-sided *p*-value of <0.05.

## 3. Results

**Figure 1:**
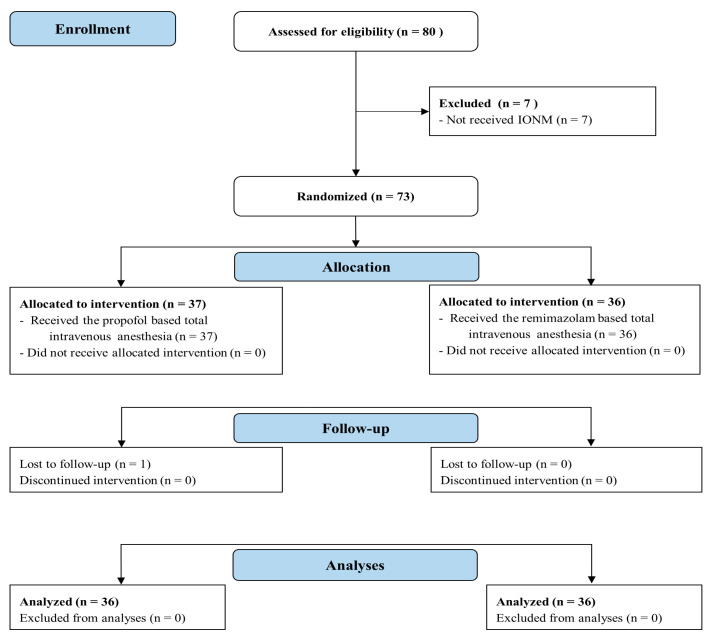
CONSORT flow diagram.

Of the 80 patients assessed for eligibility, 73 were enrolled and randomized into either group P or group R. Of these, seven patients were excluded due to their lack of IONM, and one patient was excluded owing to refusal to participate in the study process. The final analysis included 72 patients (Figure 1). The preoperative characteristics of the patients did not differ between the groups (Table 1).

**Figure 2:**
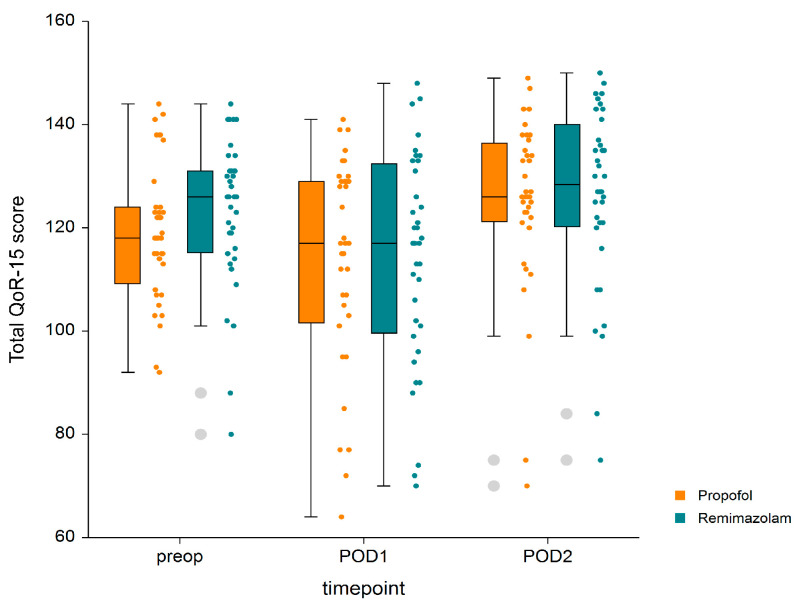
Total QoR-15 scores. Boxplots represent medians and 25% and 75% interquartile ranges. Dotted plots show the QoR-15 score distribution. Abbreviations: POD, postoperative day; preop, preoperative; QoR-15, Quality of Recovery-15 questionnaire.

Figure 2 and Supplementary Figure S2 (Supplementary Digital Material) present the global QoR-15 scores and their respective sub-scores measured at three time points: preoperatively, on POD1, and on POD2. No significant differences were observed in the total QoR-15 scores measured on POD1 between the two groups (114 [±20.1] vs. 112 [±23.1] points, respectively, *p* = 0.691). However, the total QoR-15 scores on POD1 in both groups significantly decreased compared to the preoperative scores (*p* = 0.032 in group P, *p* = 0.007 in group R). Furthermore, the total QoR-15 scores on POD 2 improved significantly in both groups compared to POD1 (*p* < 0.001 for each) (Figure 2). There were no significant interaction between group and time in the total QoR-15 scores and the five dimensions of QoR-15 in either group (Supplementary Digital Material: Supplementary Figures S3 and S4).

**Figure 3:**
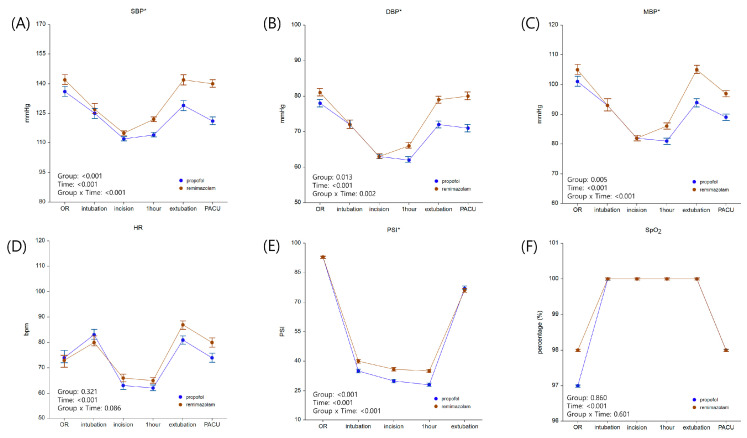
Perioperative data: (**A**) SBP, (**B**) DBP, (**C**) MBP, (**D**) HR, (**E**) PSI, and (**F**) SpO_2_. Values are presented as estimated mean and standard error. * represents variables with significant interactions between group and time after analysis using linear mixed model. Abbreviations: bpm, beats per minute; DBP, diastolic blood pressure; HR, heart rate; MBP, mean blood pressure; OR, operating room; PACU, post-anesthetic care unit; PSI, patient state index; SBP, systolic blood pressure; SpO_2_, peripheral oxygen saturation.

Table 2 presents the intraoperative parameters. Notably, the total remifentanil consumption was significantly higher in group R (2066.8 [680.8] µg) compared to group P (1452.4 [410.2] µg, *p* < 0.001), while the total amount of phenylephrine was significantly lower in group R (286.1 [442.0] µg) compared to group P (1705.6 [1877.0] µg, *p* < 0.001). Figure 3 depicts the perioperative hemodynamic status and intraoperative PSI values. Patients who received remimazolam displayed more stable blood pressure over time than those who received propofol, with the difference being significant (*p* < 0.001 for group R).

Table 3 presents an overview of the profile of postoperative recovery. The time to reach a PSI score of >50 points and obey commands was significantly shorter in group R than in group P (366.1 [206.9] s vs. 609.9 [243.2], respectively, *p* < 0.001; and 594.7 [197.9] s vs. 714.0 [254.4], respectively, *p* = 0.030). In the PACU, the NRS score, which indicates the degree of pain intensity in the resting state, was significantly higher in group P than in group R (3.0 [0.0] vs. 2.8 [0.5], respectively, *p* = 0.009). There was no significant difference in the requirement of rescue analgesics and antiemetics between the two groups in the PACU. One patient in group P experienced postoperative adverse events due to a wound. Nevertheless, no residual complications were observed among the study population until hospital discharge and no significant differences were observed in the postoperative adverse events and length of hospital stay between the two groups.

## 4. Discussion

In this present study, we compared the overall functional recovery in patients who underwent spine surgery with IONM with either remimazolam- or propofol-based TIVA. Generally, IONM is safe and sensitive for detecting intraoperative nerve injuries during spine surgeries [25]. However, it is inevitably affected by the anesthetic method or agent used; efforts are being made to determine optimal anesthetic management, including drugs that have minimal or no effects on neurophysiological monitoring [26]. Accordingly, TIVA with propofol and remifentanil is frequently used to optimize IONM when compared to inhalation anesthesia [27]. To the best of our knowledge, our study is the first to report the QoR in patients following spine surgery requiring IONM. In our study, on POD1, the total QoR-15 score for group R was 112, whereas that for group P was 114. Moreover, no significant differences emerged in the group–time interaction effect between groups R and P. In an assessment of the five QoR dimensions, group R failed to demonstrate markedly superior scores compared with group P. However, an intriguing observation surfaced in outcomes other than QoR; patients who received remimazolam exhibited more stable intraoperative blood pressure, shorter awakening time, and less pain intensity in the recovery period than patients who received propofol.

In a previous study that compared remimazolam-based TIVA to propofol-based TIVA in patients who underwent thyroidectomy, similar postoperative global QoR-15 scores along with more stable hemodynamic fluctuations were observed [7]. This suggests a favorable profile for remimazolam in thyroid surgery, aligned with its effectiveness and hemodynamic benefits. In contrast, another study in the realm of minor urological surgery found that the total QoR-15 scores were significantly lower for remimazolam-based TIVA than for propofol-based TIVA [28]. These contrasting outcomes underscore the importance of considering specific surgical contexts when evaluating the effects of anesthetic agents on QoR. The diverse nature of surgeries may contribute to variations in the observed effects of remimazolam, emphasizing the need for tailored approaches based on the surgical procedure.

Considering that the QoR-15 is a robustly verified multidimensional patient-reported outcome assessment tool, employing a survey is essential for evaluating the quality of recovery after surgery, tailored to the specific surgical procedure [10,11,29]. The ability of a tool to identify and estimate meaningful changes clinically is fundamental for predicting patient prognosis in the context of health conditions [30]. In particular, the QoR-15 has undergone validation across various linguistic and cultural settings, including validation studies conducted with Korean patients [31]. The concept of an acceptable patient symptom status further enhances its utility by representing the minimum definite score on the scale of health that a patient perceives as indicative of good status [7,32].

Although remimazolam lacked superiority in terms of QoR in this study, it has several advantages with TIVA use. Remimazolam helps in maintaining hemodynamic stability during surgery [2]. It induces a dose-dependent increase in intracellular calcium levels, particularly through its effect on the G-protein-coupled receptor–inositol 1,4,5-triphosphate pathway [33]. Consistent with this mechanism, we found that group R had significantly lower phenylephrine consumption compared with group P, confirming the hemodynamic stability observed in previous investigations in minimizing intraoperative vasopressor requirements [7,34,35]. Moreover, remimazolam exhibited improved awakening times, even when accounting for the limited use of a reversal agent. This advantage is especially significant in spinesurgery where immediate neurological examination is imperative.

Remifentanil consumption during surgery was notably higher in group R compared to group P. Despite concerns about opioid-induced hyperalgesia (OIH), our study showed that group R experienced less pain intensity compared with group P while resting in the PACU. This discrepancy is likely attributed to the effects of remimazolam on GABA receptors, potentially affecting pain perception [36]. While one might anticipate delayed recovery time, increased pain intensity due to OIH, and frequent use of rescue analgesics in group R, which received higher doses of remifentanil, the remimazolam group remarkably exhibited more favorable outcomes in these aspects in this study. In addition, there was no discernible difference in postoperative nausea and vomiting (PONV) compared to propofol.

This study has several limitations that warrant consideration. First, we did not compare the intraoperative neurophysiological signals between the two groups, owing to our primary focus on functional recovery and the immediate postoperative impact of anesthesia. Although group R exhibited statistically superior outcomes regarding hemodynamic stability, recovery time, and postoperative pain compared to group P, the absence of a comparison of neurophysiological signals limits the comprehensiveness of our findings. Second, the depth of anesthesia during surgery was applied uniformly without considering differences in drugs. Benzodiazepines and propofol have different effects on an electroencephalogram, and the index of existing anesthesia monitoring equipment does not include a remimazolam-based algorithm. Therefore, relying only on the comparison of PSI values does not provide meaningful clinical insight [2]. Third, comparing the consumption of agents during surgery between remimazolam and propofol, the cost of remimazolam-based TIVA would obviously be more expensive than propofol-based TIVA in most countries. Nevertheless, its use should be worth the increased cost because, as already mentioned, there is an antidote, there is no possibility of propofol infusion syndrome, and blood pressure is more stable during surgery.

Lastly, the rather small number of participants may limit the generalizability of the results. While most minimal clinically important differences (MCIDs) reported in various studies fell below 8 points for the total QoR-15 score [7,28], our observed 13-point difference could signify substantial clinical improvement in functional recovery beyond the MCID criteria [24].

## 5. Conclusions

Our findings revealed that remimazolam was not statistically superior to propofol in terms of total postoperative QoR-15 scores. Nevertheless, remimazolam demonstrated proficiency in maintaining stable hemodynamics throughout the surgical procedure. Additionally, patients who received remimazolam-based TIVA reported better recovery regarding postoperative pain in the PACU and recovered faster from anesthesia than patients who received propofol-based TIVA. These results indicate the advantages of remimazolam, particularly in the immediate postoperative recovery period following spine surgery requiring IONM, and suggest its potential as an alternative to propofol. Particularly, for high-risk patients, such as those for whom propofol is not advisable or those who are hemodynamically unstable, remimazolam may provide a safe anesthetic environment. We expect that further research with a larger study population and the incorporation of IONM data into analyses would address some of the limitations of this current study.

## Figures and Tables

**Table 1:** Patient characteristics.

	Group P (n = 36)	Group R (n = 36)	Standardized Difference
Sex (male/female)	23/13	19/17	0.227
Age (years)	50.3 [33–66]	54.2 [27–66]	0.447
Height (cm)	168.0 (6.2)	165.8 (9.5)	0.272
Weight (kg)	65.5 (11.6)	65.0 (9.4)	0.050
Diagnosis			
Stenosis	17 (47.2%)	14 (38.9%)	0.169
Disc herniation	8 (22.2%)	9 (25.0%)	0.065
Cervical myelopathy	3 (8.3%)	7 (19.4)	0.326
OPLL	3 (8.3%)	3 (8.3%)	0
Others	5 (13.9%)	3 (8.3%)	0.178
Past medical history			
Smoking	15 (41.7%)	9 (25.0%)	0.359
Steroid use	3 (8.3%)	2 (5.6%)	0.109
Psychiatric medication	3 (8.3%)	4 (11.1%)	0.094
Comorbidities			
Hypertension	9 (25.0%)	6 (16.7%)	0.206
DM	4 (11.1%)	4 (11.1%)	0
Cardiac	0	3 (8.3%)	0.426
Respiratory	4 (11.1%)	9 (13.9%)	0.084
Others	6 (16.7%)	4 (11.1%)	0.161

Values are expressed as number of patients, means (standard deviations) (except age: mean [range]), or numbers (percentiles). Abbreviations: DM, diabetes mellitus; OPLL, ossification of posterior longitudinal ligament; P, propofol; R, remimazolam.

**Table 2:** Parameters related to anesthesia and surgery.

	Group P (n = 36)	Group R (n = 36)	*p*-Value
Time to loss of consciousness (s)	78.1 (30.5)	77.1 (14.5)	0.868
Time to PSI < 50 (s)	146.1 (68.3)	156.8 (44.2)	0.433
Duration of anesthesia (min)	174.1 (43.4)	176.5 (48.6)	0.831
Duration of operation (min)	117.6 (36.0)	118.2 (41.4)	0.949
Total input (mL)	1350.7 (543.9)	1330.6 (475.1)	0.809
Total output (mL)	440.0 (75.0, 1075.0)	400.0 (100.0, 600.0)	0.368
Total consumption of remifentanil (μg) *	1425.4 (410.2)	2066.8 (680.8)	<0.001
Total amount of phenylephrine (μg) *	1200.0 (340.0, 2420.0)	185.0 (0.0, 360.0)	<0.001
Sugammadex	0	1 (2.8%)	>0.999
Flumazenil	0	2 (5.6%)	>0.999

Values are expressed as means (±standard deviations), median (interquartile range), or numbers (percentiles). * *p* < 0.05. Abbreviations: P, propofol; PSI, patient state index; R, remimazolam.

**Table 3:** Postoperative recovery profiles.

	Group P (n = 36)	Group R (n = 36)	*p*-Value
After anesthesia completion			
Time to obey command (s) *	714.0 (254.4)	594.7 (197.9)	0.030
Time to PSI > 50 (s) *	609.9 (243.2)	366.1 (206.9)	<0.001
Time to extubation (s)	785.9 (268.6)	702.1 (239.2)	0.167
Adverse events related extubation	0	2 (5.6%)	0.493
In PACU			
NRS of pain intensity (resting) *	3.0 (0.0)	2.8 (0.5)	0.009
NRS of pain intensity (acting)	5.2 (0.7)	4.8 (0.9)	0.086
Requested rescue analgesics	9 (25%)	7 (19.4%)	0.571
Morphine equivalent dose of analgesics (mg)	1(2.2)	1.3(2.2)	0.688
Requested antiemetics	0 (0%)	0 (0%)	>0.999
Duration of PACU stay (min)	43.1 (21.5)	43.3 (17.9)	0.953
Postoperative 1–6 h			
NRS of pain intensity (at rest)	3.4 (0.5)	3.3 (0.7)	0.703
NRS of pain intensity (during movement)	5.3 (0.7)	5.1 (0.7)	0.130
Requested rescue analgesics	2 (5.6%)	5 (13.9%)	0.429
Morphine equivalent dose of analgesics (mg)	0.7(1.8)	0.3(1.2)	0.239
Requested antiemetics	1 (2.8%)	0 (0%)	0.239
Postoperative 6–24 h			
NRS of pain intensity (at rest)	1.9 (0.7)	1.9 (0.7)	0.748
NRS of pain intensity (during movement)	3.8 (1.4)	3.7 (1.0)	0.848
Requested rescue analgesics	3 (8.3%)	7 (19.4%)	0.173
Morphine equivalent dose of analgesics (mg)	1(2)	0.4(1.4)	0.178
Requested antiemetics	3 (8.3%)	2 (5.6%)	>0.999
Postoperative 24–48 h			
NRS of pain intensity (at rest)	1.5 (0.6)	1.3 (0.6)	0.169
NRS of pain intensity (during movement)	3.4 (0.9)	3.1 (0.6)	0.105
Requested rescue analgesics	3 (8.3%)	4 (11.1%)	>0.999
Morphine equivalent dose of analgesics (mg)	0.6(1.6)	0.4(1.4)	0.696
Requested antiemetics	0 (0%)	0 (0%)	>0.999
PCA withdrawal due to PONV	12 (33.3%)	13 (36.1%)	0.805
Postoperative adverse events	1 (2.8%)	0	>0.999
Postoperative hospital stay duration	4.4 (2.4)	4.2 (1.9)	0.632

Values are expressed as numbers (percentiles) or means (standard deviations). * *p* < 0.05. Abbreviations: NRS, numeric rating scale; P, propofol; PACU, post-anesthesia care unit; PCA, patient-controlled analgesia; PONV, postoperative nausea and vomiting; PSI, patient state index; R, remimazolam.

## Data Availability

The data associated with the paper are not publicly available, but are available from the corresponding author upon reasonable request.

## Supplementary Materials

The following supporting information can be downloaded at: https://www.mdpi.com/article/10.3390/jpm14040382/s1, Supplementary Figure S1. Quality of Recovery-15 (QoR-15) questionnaire. Supplementary Figure S2. Boxplots for the five dimensions of the QoR-15. Boxplots show the median 25% and 75% interquartile ranges. (A) physical comfort, (B) emotional state, (C) psychological support, (D) physical independence, and (E) pain. POD, postoperative day; preop, preoperative; QoR-15, Quality of Recovery-15 questionnaire. Supplementary Figure S3. Line chart of the total QoR-15 scores. Values are presented as the estimated mean and standard error. POD, postoperative day; preop, preoperative; QoR-15, Quality of Recovery-15 questionnaire. Supplementary Figure S4. Line charts for the five dimensions of the QoR-15. Values are presented as the estimated mean and standard error. (A) physical comfort, (B) emotional state, (C) psychological support, (D) physical independence, and (E) pain. POD, postoperative day; preop, preoperative; QoR-15, Quality of Recovery-15 questionnaire.
