# Supplementary figures and images for: Quality of Postoperative Recovery in Total Intravenous Anesthesia between Remimazolam and Propofol for Intraoperative Neurophysiological Monitoring: A Prospective Double-Blind Randomized Controlled Trial
Source: J Pers Med. 2024 Apr 2;14(4):382. doi: 10.3390/jpm14040382 (PMC11051443; doi:10.3390/jpm14040382)

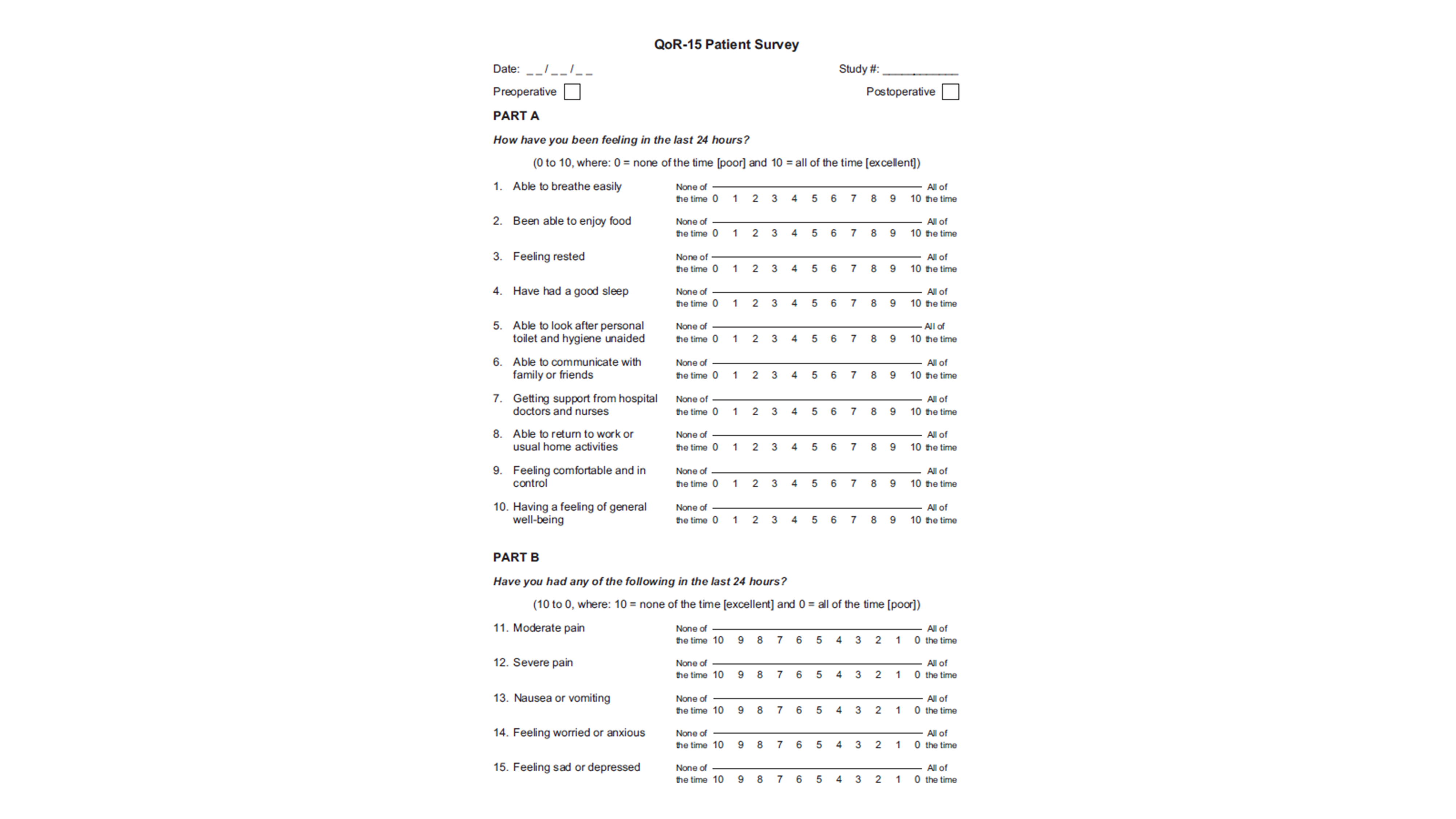

Supplement: Supplementary file 1 [file jpm-14-00382-s001.zip › suppl fig. 1 QoR-15 patient survey.tif]

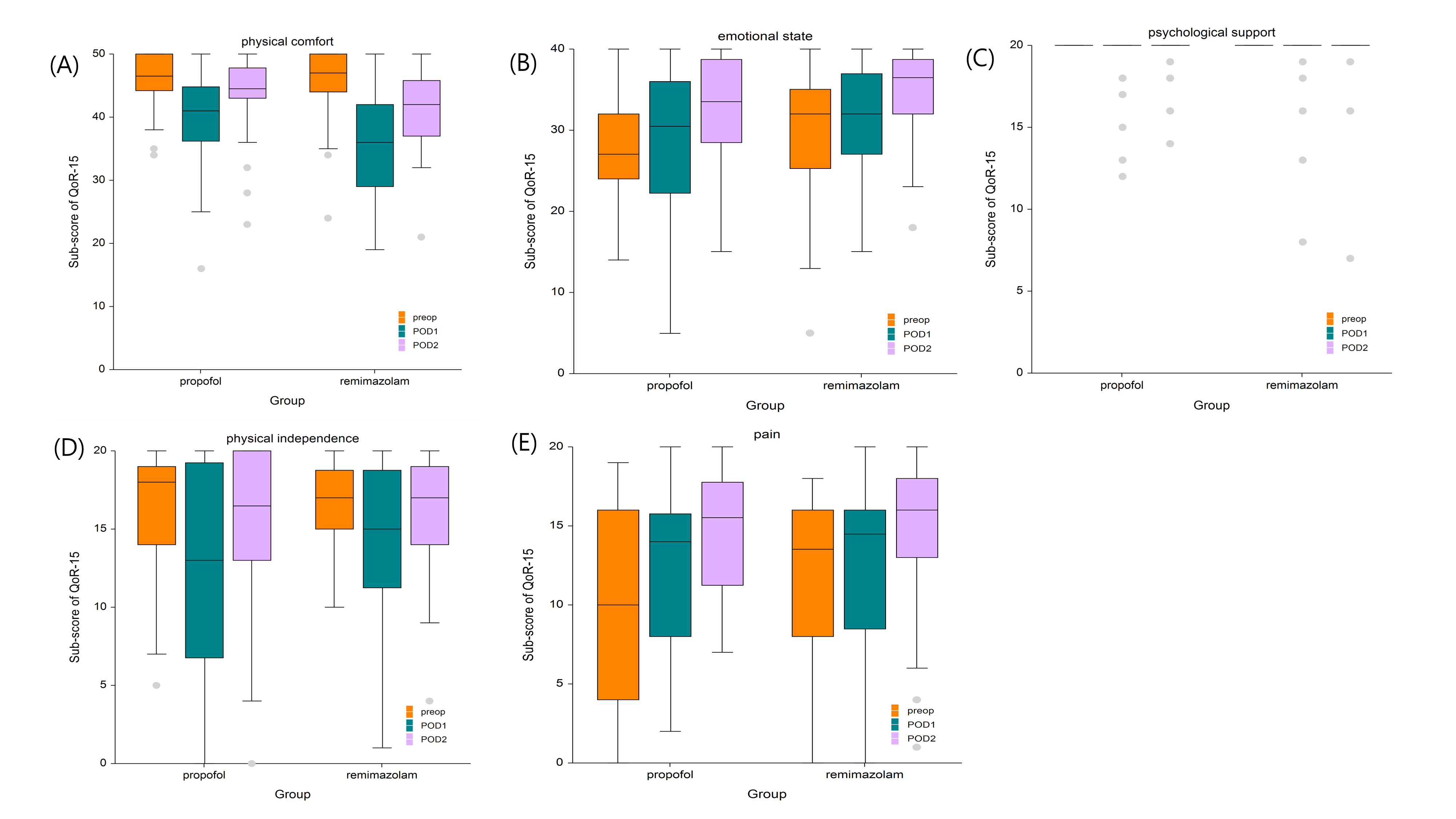

Supplement: Supplementary file 1 [file jpm-14-00382-s001.zip › suppl fig. 2 Boxplots of QoR-15 5 dimensions.tif]

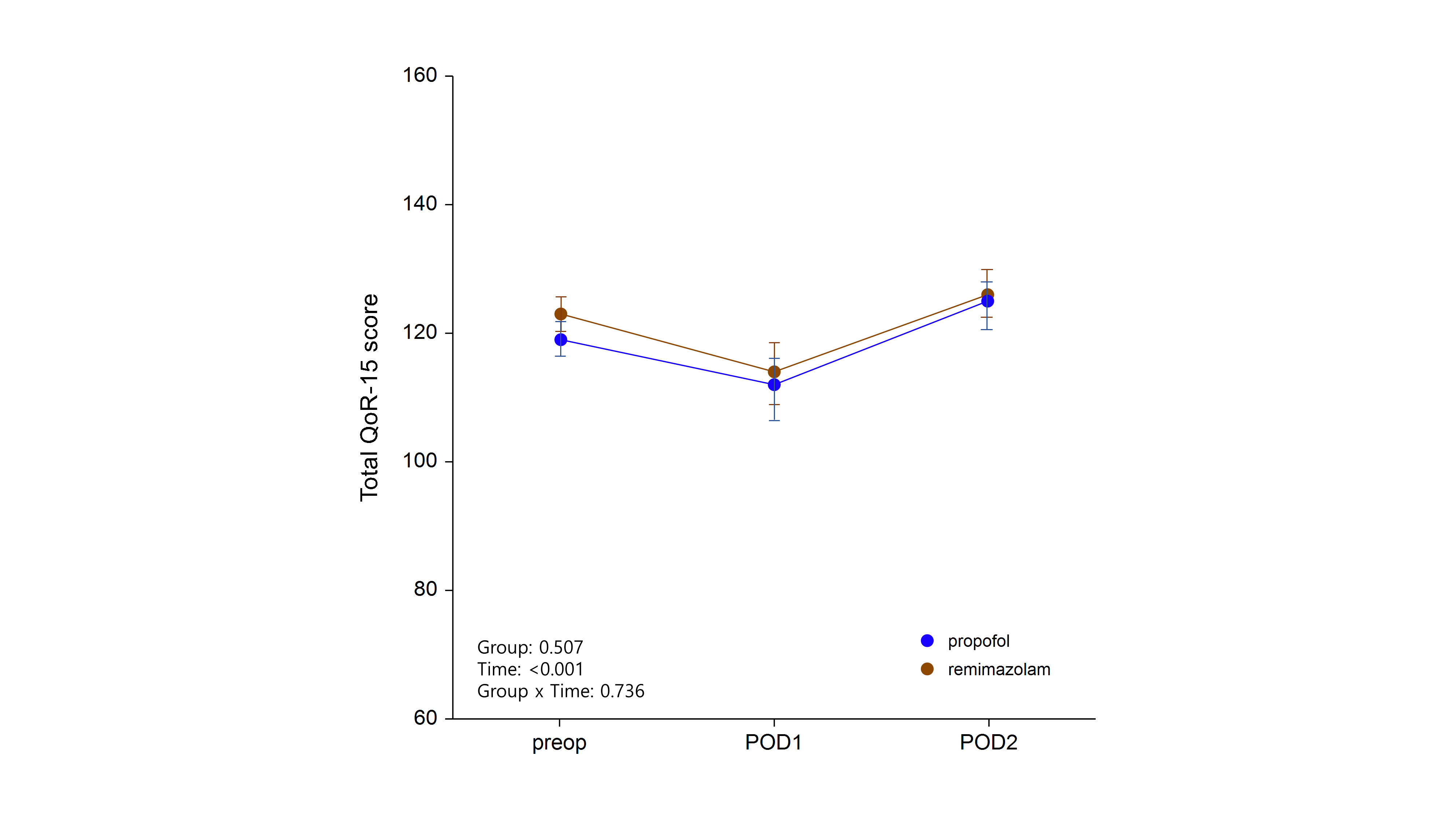

Supplement: Supplementary file 1 [file jpm-14-00382-s001.zip › suppl fig. 3 Line chart of total QoR-15 score.tif]

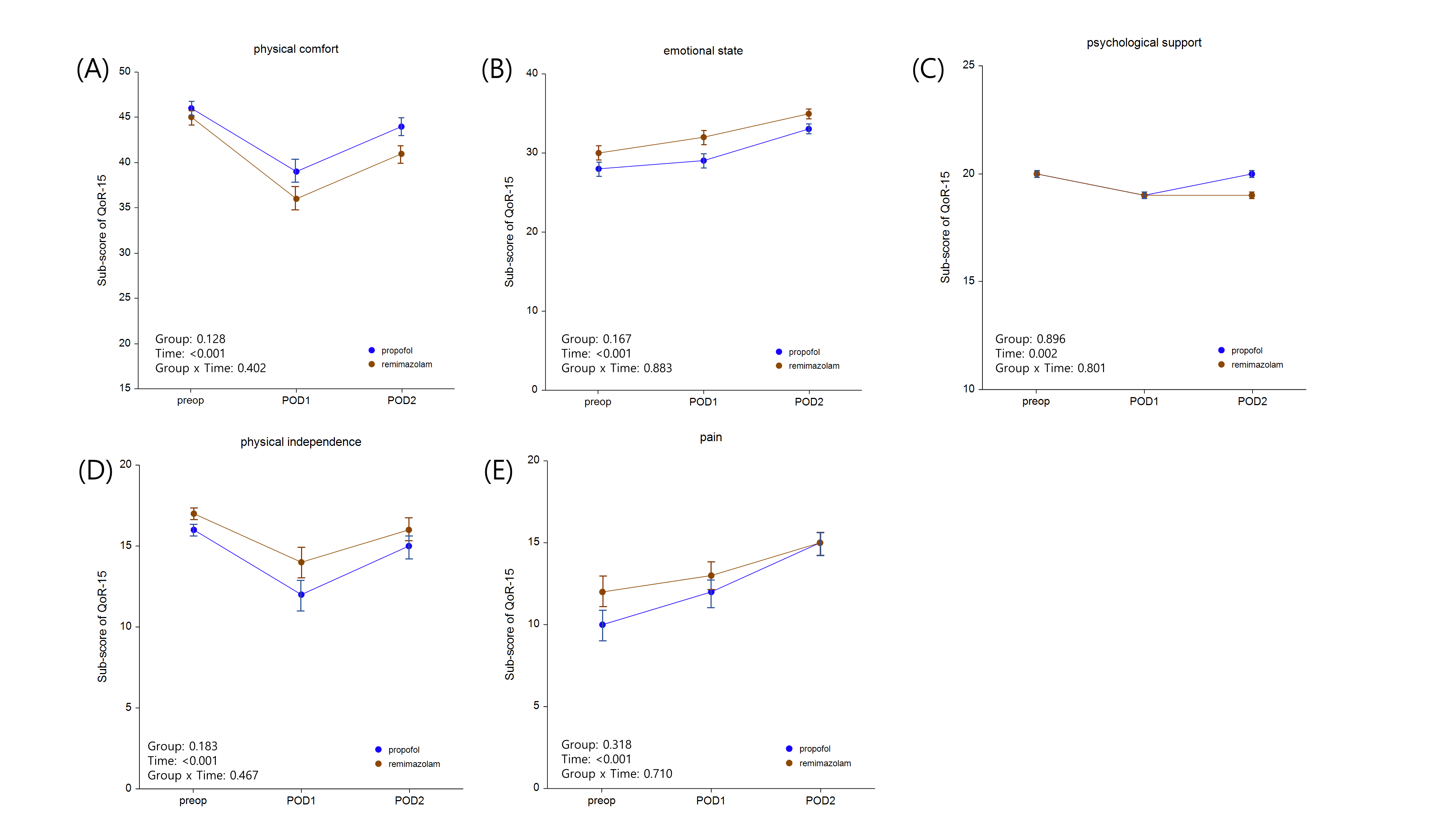

Supplement: Supplementary file 1 [file jpm-14-00382-s001.zip › suppl fig. 4 Line chart of QoR-15 5 dimensions.tif]
